# Patterns of lower limb muscular activity and joint moments during directional efforts using a static dynamometer

**DOI:** 10.1186/s42490-019-0035-7

**Published:** 2020-01-08

**Authors:** Mathieu Lalumiere, Cloé Villeneuve, Cassandra Bellavance, Michel Goyette, Daniel Bourbonnais

**Affiliations:** 1 School of Rehabilitation, University de Montréal, C.P. 6128, succursale Centre-ville, Montreal, H3C 3J7 Canada; 2 Centre for Interdisciplinary Research in Rehabilitation of Greater Montreal (CRIR), Montreal, Canada

**Keywords:** *(3–10)* dynamometer, Lower limb, Rehabilitation, Electromyography, Muscle strength, Gait

## Abstract

**Background:**

Strength and coordination of lower muscle groups typically identified in healthy subjects are two prerequisites to performing functional activities. These physical qualities can be impaired following a neurological insult. A static dynamometer apparatus that measures lower limb joint moments during directional efforts at the foot was developed to recruit different patterns of muscular activity. The objectives of the present study were to 1) validate joint moments estimated by the apparatus, and 2) to characterize lower limb joint moments and muscular activity patterns of healthy subjects during progressive static efforts. Subjects were seated in a semi-reclined position with one foot attached to a force platform interfaced with a laboratory computer. Forces and moments exerted under the foot were computed using inverse dynamics, allowing for the estimation of lower limb joint moments.

To achieve the study’s first objective, joint moments were validated by comparing moments of various magnitudes of force applied by turnbuckles on an instrumented leg equipped with strain gauges with those estimated by the apparatus. Concurrent validity and agreement were assessed using Pearson correlation coefficients and Bland and Altman analysis, respectively. For the second objective, joint moments and muscular activity were characterized for five healthy subjects while exerting progressive effort in eight sagittal directions. Lower limb joint moments were estimated during directional efforts using inverse dynamics. Muscular activity of eight muscles of the lower limb was recorded using surface electrodes and further analyzed using normalized root mean square data.

**Results:**

The joint moments estimated with the instrumented leg were correlated (r > 0.999) with those measured by the dynamometer. Limits of agreement ranged between 8.5 and 19.2% of the average joint moment calculated by both devices. During progressive efforts on the apparatus, joint moments and patterns of muscular activity were specific to the direction of effort. Patterns of muscular activity in four directions were similar to activation patterns reported in the literature for specific portions of gait cycle.

**Conclusion:**

This apparatus provides valid joint moments exerted at the lower limbs. It is suggested that this methodology be used to recruit muscular activity patterns impaired in neurological populations.

## Background

Muscle weakness, defined as the inadequate capacity to generate normal levels of force [1], is a common deficit following a neurological insult such as stroke [2]. Several correlational studies have found a positive relationship between lower limb (LL) muscle strength and functional activities such as walking, stair climbing and sit-to-stand transfers in this population [3–5]. Systematic reviews have provided evidence that progressive resistive training increases muscle strength in stroke patients [6–8]. However, these gains may not translate into improved functional performance [6]. In a recent meta-analysis, it was highlighted that of 12 studies in which more than 80% of the experimental intervention was dedicated to LL strength training, only three studies reported statistically significant improvements in walking gait velocity (0.9 to 1.5 m/s) in either subacute (*n* = 1) or chronic stroke patients (*n* = 2) [7]. Interestingly, the training programs used in three studies focused on multi-articular strengthening exercises, which suggests that improved functional performance could be related to the reinforcement of multi-articular muscles. In another recent meta-analysis, it was shown that the use of an isokinetic dynamometer is a suitable strategy for improving multi-articular muscle strength and functional mobility during walking in stroke patients [8].

**Fig. 1:**
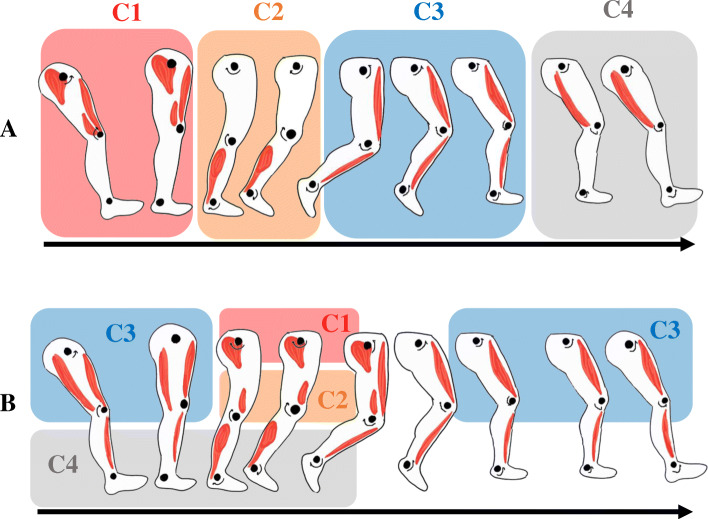
**a**) Modules of muscular activity identified by matrix factorization during gait. From 0 to 12% of the gait cycle (C1), gluteus medius, vastus medialis and rectus femoris are activated and provide body support and decelerate forward motion during early stance. From 30 to 50% of the gait cycle (C2), medial gastrocnemius and soleus are activated and provide body support and forward propulsion during stance. From 62 to 75% of the gait cycle (C3), tibialis anterior and rectus femoris ensure limb clearance during the early swing phase. From 87 to 100% of the walking cycle (C4), the semitendinosus and biceps femoris decelerate the limb during the late swing phase. **b**) Merging of the four muscular modules after a stroke. Compared to healthy subjects, the modules are modified during the gait cycle. Based on data presented by Clark et al. 2010

Moreover, it has been suggested that strength deficits in LL muscles are not the only limiting factors to improving gait in stroke patients. Lack of activation and synchronization of muscles required for walking also plays a role [9]. Coordination of the lower segments during gait is a complex task requiring specific joint biomechanics and precise co-activation of several muscles [10]. Various studies have shown that electromyographic (EMG) activity recorded during normal human gait is reproduced as a linear combination of four basic patterns or modules (C1, C2, C3 and C4, Fig. 1.a.) [11, 12]. Following a neurological insult such as stroke, fewer modules are required to account for muscle activation during walking (Fig. 1.b.), suggesting a reduction in overall motor complexity [11] correlated with the degree of motor impairment (i.e., step length asymmetry and slower gait speed) [12]. Patterns of muscular activity has previously been studied and compared to gait during functional movements such as cycling [13]. However, no study has compared gait patterns of muscular activity to the muscular activity measured during directional efforts on a static dynamometer.

To improve LL muscle coordination during rehabilitation, it was previously shown that real-time visual feedback can be used for balance and gait training among the elderly [14] and post-stroke populations [15]. To our knowledge, no study has previously used directional visual feedback with a static dynamometer to improve muscle strength and coordination based on combination of moments of force at several joints of the LL. An experimental set-up was adapted from a previous training program that used the force feedback of a static dynamometer to improve mobility among stroke patients [16]. This approach essentially uses force feedback to exert static efforts in multiple directions and recruits different patterns of muscular activity at the LL. Using a static apparatus to regain the ability to perform a dynamic task is justified by the general concept of gait training prerequisites, such as LL muscle coordination for body support and forward progression, without the simultaneous use of core muscles for balance control [17]. Therefore, a progressive, static dynamometer training program focused on the recruitment of gait-related muscular activity patterns could be used as a restorative approach in addition to conventional task-specific training to improve mobility during intensive rehabilitation.

As a first step, we aimed to develop a methodology to characterize muscle activation patterns in healthy subjects during distal directional efforts of the foot eliciting multi-articular joint moments in the LLs. The specific objectives of the present methodological study were 1) to validate the multi-articular joint moments estimated using this methodology by comparing moments of various magnitudes of force applied by turnbuckles on an instrumented leg with those estimated by the apparatus, and 2) to characterize LL joint moments and muscle activity of healthy subjects while exerting progressive static effort on the apparatus in multiple directions to estimate the feasibility of the methodology for future clinical studies. It was hypothesized that 1) the joint moments estimated using the instrumented leg would correlate and agree with those measured by the apparatus, and that 2) lower limb joint moments and patterns of muscular activity on the apparatus would be modified according to the direction of effort and correlate to the muscular activation patterns previously observed during gait.

## Methods

### Description of the instrumented set-up

**Fig. 2:**
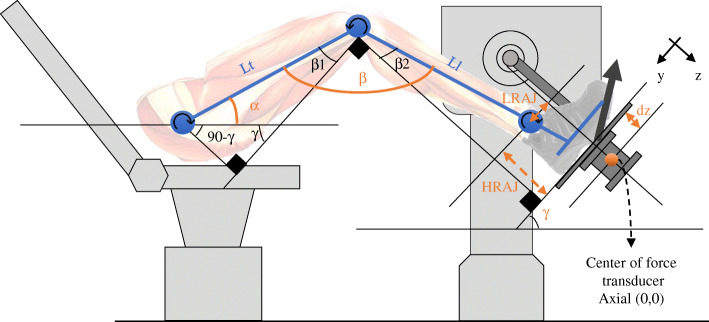
The subject’s foot is firmly secured on a force transducer interfaced with a laboratory computer. The location of the center of pressure in the Y axis is monitored in real time. By measuring the different angles (α, β,γ), the different lever arms (Ll, Lt, LRAJ, HRJ), and the force vector, the joint moments exerted at the different joints can be calculated

The apparatus used in this research consisted of a static dynamometer (Biodex Medical Systems, NewYork, USA) with an adjustable chair on which subjects sat leaning back with their foot attached to a force platform (AMTI model MC3–1000, Advanced Manufacturing Technology Inc., Massachusetts, USA) (Fig. 2). This experimental set-up allowed for the measurement of vertical and horizontal forces (Fy and Fz) and moments of force (Mx, My) exerted under the foot at the center of the force transducer. These kinetic values were digitized from the output of strain gauge amplifiers using an acquisition card and fed into a computer at a frequency of 100 Hz. A software (Labview; National Instruments, Texas, USA) was developed to calculate the joint moments at the hip, knee and ankle by inverse dynamics using the data collected from the AMTI force platform and the subjects’ anthropometric information.

The height and position of the chair were adjusted to ensure that the foot was positioned at 55 degrees (γ angle) from the horizontal plane, with 20 degrees of hip flexion (α angle) and 125 degrees of knee flexion (β angle) (Fig. 2). This position was chosen since it largely corresponds to the mean values of joint angle changes during walking [18], allowing for the exertion of positive and negative moments at each joint. All three angles were validated using a goniometer.

The angles (α, β and γ) were entered into the software as well as the values for the different lever arm distances (Lt, Ll, LRAj, HRAj) measured using a measuring tape. The distance (d_z_) between the center of the AMTI transducer and the plate was provided by the manufacturer.

Based on the measurements of the lever arms (Lt, Ll, HRAJ, LRAJ, dz) and angles **(**α, β,γ) illustrated in Fig. 2, it was possible to calculate the distance between the AMTI force transducer center of reference and the articular center of rotation of the hip (*rhj*), knee (*rkj*) and ankle joint (*raj*) in the y and z directions using eqs. 1–6, where β1 = γ - α and β2 = β − 90- γ + α.

1 $$ {raj}_y=\mathrm{LRAJ} $$

2 $$ {raj}_z=\mathrm{HRAJ}+{d}_z $$

3 $$ {rkj}_y=\mathrm{LRAJ}+\mathrm{Ll}\ast \sin \left(\upbeta 2\right) $$

4 $$ {rkj}_z=\mathrm{HRAJ}+{d}_z-\mathrm{Ll}\ast \cos \left(\upbeta 2\right) $$

5 $$ {rhj}_y=\mathrm{LRAJ}+\mathrm{Ll}\ast \sin \left(\upbeta 2\right)+ Lt\ast \cos \left(\upbeta 1\right) $$

6 $$ {rhj}_z=\mathrm{HRAJ}+{d}_z-\mathrm{Ll}\ast \cos \left(\upbeta 2\right)- Lt\ast \sin \left(\upbeta 1\right) $$

By measuring the location of the center of pressure exerted on the AMTI platform in relation to the y axis (COP_y_), by calculating the direction of the force vectors applied at AMTI force platform located at the end of the LL (F_y_,F_z_) and by using *rhj, rkj*, *raj*, it was possible to calculate the different joint moments exerted at the hip (Mh), knee (Mk) and ankle (Ma) (Eq. 7).

7 $$ \left[\begin{array}{c} Ma\\ {} Mk\\ {} Mh\end{array}\right]=\left[\begin{array}{l}{COP}_y-{raj}_{\mathrm{y}}-\left({raj}_z-{d}_z\right)\\ {}{COP}_y-{rkj}_y-\left({rkj}_z-{d}_z\right)\\ {}{COP}_y-{rhj}_y-\left({rhj}_z-{d}_z\right)\end{array}\right]\times \left[\begin{array}{c}{F}_z\\ {}{F}_y\end{array}\right] $$

### Validation of the joint moments

**Fig. 3:**
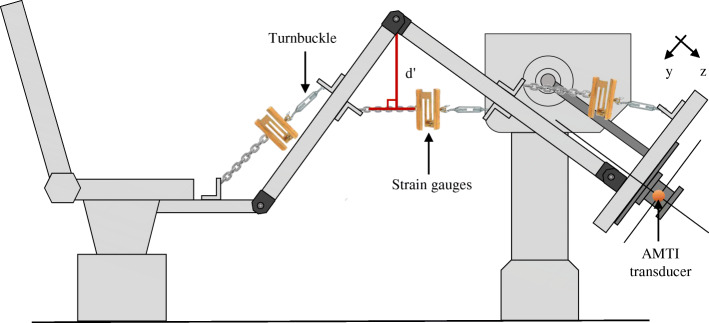
Experimental set-up for measuring moments exerted at the hip (Mh), knee (Mk) and ankle (Ma) from the strain gauges force and lever arm (d’). A turnbuckle was used to induce tension in the cable measured by a force transducer at one joint. The joint moment exerted was compared to the joint moment estimated by the apparatus

To validate the joint moments measured using the apparatus and the experimental methodology, an instrumented leg with three joints corresponding to the hip, knee and ankle was mounted on the AMTI transducer (Fig. 3). A cable equipped with a turnbuckle and strain gauges was tethered at each joint to simulate a muscle group. The moment from the strain gauges was calculated by modifying the tension in the cable and measuring the perpendicular distance between the cable (d’) and the center of rotation of the joint. Validation of the inverse dynamics data at the instrumented leg was done by comparing the expected moments at the hip (Mh), knee (Mk) and ankle (Ma) joints calculated from the AMTI transducer to the moments calculated from calibrated strain gauges positioned at the hip (Mh’), knee (Mk’) and ankle (Ma’) for 11 trials during which the tension was progressively increased at each joint.

The distance between each joint’s center of rotation was used to estimate the length of the thigh (Lt) and leg (Ll) of the instrumented leg by taking a static picture of the experimental set-up, processing the image data with Matlab, and extrapolating the distance between specific points using a ruler as a reference. The distance between the ankle joint articular center and the center of the sensor axial force parallel to the platform (LRAJ) and the height of the ankle joint center relative to the AMTI force platform (HRAJ) were also measured by the image-extrapolation method.

### Joint moments and muscular activity characterization

#### Participants

Five healthy subjects (1 man; 4 women) between the ages of 21 and 26 (22.8 ± 2.5 years of age), with no reported neurological conditions or musculoskeletal impairments limiting their mobility, took part in this study. The study was conducted at the Pathokinesiology Laboratory of the Centre for Interdisciplinary Rehabilitation Research of Greater Montreal (CRIR). Ethical approval was obtained from the Research Ethics Committees of the CRIR (1220–0317). The subjects received detailed information about the study prior to their participation and provided written consent.

#### Surface EMG recordings

Surface electromyography (EMG) of tibialis anterior (TA), soleus (SO), medial gastrocnemius (MG), vastus medialis (VM), rectus femoris (RF), biceps femoris (BF), semitendinosus (ST) and gluteus medius (GM) were recorded on the left (non-dominant) lower extremity using a portable telemetric system (NORAXON USA Inc., Scottsdale, Arizona; Telemyo 900) at a frequency of 1200 Hz (Hz). Self-adhesive surface electrodes (Ag/AgCl; Ambu BlueSensor M) were placed in accordance with SENIAM recommendations [19] on each muscle in a bipolar configuration with a 1 cm inter-electrode distance over the muscle belly, perpendicular to muscle fiber orientation, after each skin site was shaved and cleaned with alcohol [20]. EMG signals were visually inspected during static voluntary contractions performed against gravity and manual resistance according to a standardized protocol [21].

#### Assessment of dynamometry efforts

**Fig. 4:**
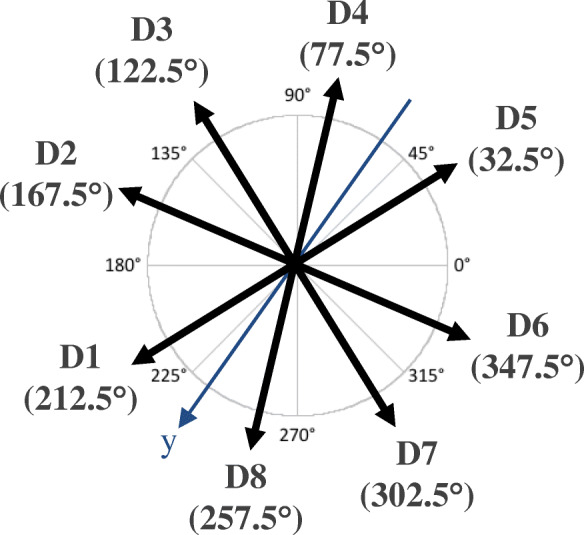
Progressive static efforts were exerted in eight directions (D1-D8) covering 360 degrees in the sagittal plane. The vector y indicates the angle of the force plate on which the foot was secured

Subjects were seated in a semi-reclined position on the static dynamometer with the non-dominant foot secured on the force platform using large Velcro straps. A force feedback cursor was displayed on a screen placed beside the subject’s side for viewing. The cursor moved horizontally or vertically in relation to the Fz and Fy force exerted at the COP of the foot. Subjects were asked to gradually move the cursor within a corridor in a specific direction for approximately two seconds at 50% of their maximal effort. The level of 50% was chosen based on preliminary tests to optimize EMG signals without excessive muscle co-contractions. Once seated and positioned on the apparatus, subjects were given two minutes to familiarize themselves with the force feedback. Subjects were then asked to exert a progressive effort ten consecutive time in eight directions, covering 360 degrees in the transverse plane of the lower extremity (Fig. 4). A one-minute break was allowed between each direction to limit muscle fatigue. The joint moments at the hip, knee and ankle were calculated but not displayed. Subjects were asked to control only the direction and magnitude of the force vector they produced.

#### Data processing

The EMG recordings were filtered using a fourth-order Butterworth zero-lag bandpass filter with cut-off frequencies set at 10 and 400 Hz. The EMG values were subsequently root mean squared (RMS) with a centered 250 msec moving window to finally generate linear envelopes [22].

Kinetic and EMG data were collected for 10 dynamometry cycles (push to end of push) and an average of 5 consecutive cycles according to the minimal EMG RMS variation coefficient was retained for analysis. RMS values were amplitude normalized from their peak values and expressed between 0 to 1 to reduce inter- and intra-subject variability [23].

Joint moments at the ankle, knee and hip, and EMG envelopes were time normalized (0 to 100% in 1% increments) relative to each push cycle and averaged together. An average of the 90–100% cycle phase (end of push) was calculated for the joint moments and EMG normalized RMS for each subject.

#### Statistics

The mean and standard deviation (SD) moments for each joint measured by the strain gauges and the moments estimated by the AMTI force platform during validation were calculated across all trials. To assess concurrent validity between the expected moments estimated by the AMTI transducer and those calculated by the calibrated strain gauges across each joint, root mean square error (RMSE), Pearson correlation (r) and determination coefficients (R^2^) were used. Bland and Altman plots and limits of agreement with confidence intervals (CI) were calculated for each of the three joints to determine the level of agreement between the moments calculated by our apparatus and by the strain gauges [24, 25]. The mean and SD of each joint’s moment and EMG values during the end of the dynamometry cycle were calculated across all subjects for all eight directions.

To assess and quantify the similarity between the normalized values of the eight muscle groups measured across the directions on the static dynamometer and the weight value of the muscular synergies previously measured during gait in a group of healthy individuals [12], cosine similarity was used and the highest value was selected for each synergy [26]. Muscle weightings were categorized as similar when the cosine similarities were over 0.71 (*p* < 0.01). All statistical analyses were performed using SPSS v.24 (SPSS Inc., Chicago, IL, USA). The *p-values* were set at 0.05.

## Results

### Validation of the instrumented set-up

The mean and standard deviation (SD) moments calculated by the strain gauges at the ankle, knee and hip of the articulated metal leg as well as the moments estimated by the AMTI force platform are presented in Table 1. The RMSE was found to be lower than 1 **N·m** for the three joints. The Pearson correlation between the calculated moments and the estimated moments was higher than 0.9994 (*p* < 0.001) for the three joints.

**Table 1:** Mean (SD) joint moments measured by the strain gauges and estimated by the AMTI force platform

Joint moment	Strain gauges	AMTI	∆ (%)	RMSE (N·m)	r (***p*** < 0.001)
Ankle dorsiflexion	**7.77 (3.92)**	**7.02 (4.23)**	**−9.6**	**0.80**	**0.9999**
Knee flexion	**12.02 (6.49)**	**11.42 (6.28)**	**−5.0**	**0.64**	**0.9999**
Hip flexion	**6.76 (3.71)**	**6.89 (3.75)**	**1.9**	**0.19**	**0.9994**

The mean difference (**∆) expressed as a %, root mean square error (RMSE) expressed in N·m and Pearson correlation coefficient (r) were calculated between both sets of measurements**

**Fig. 5:**
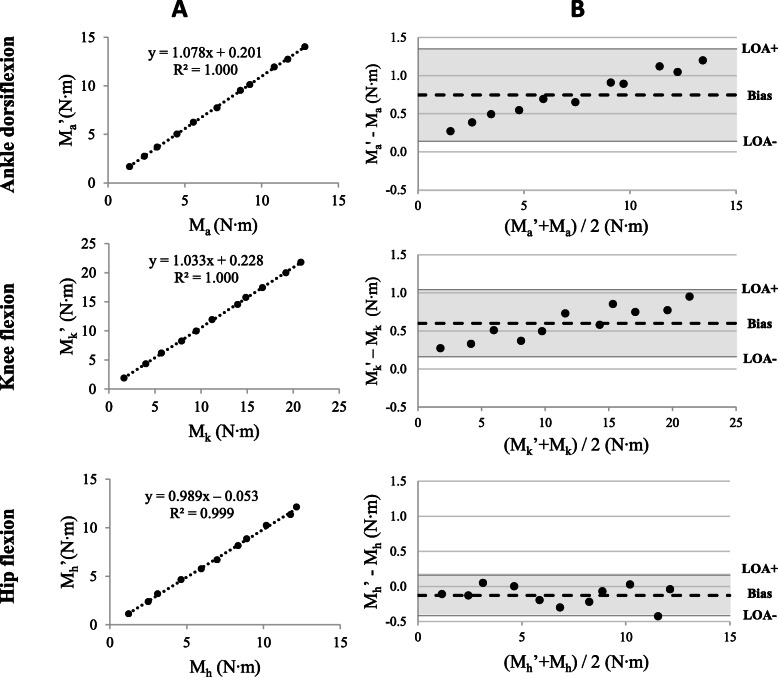
**a**) The regression line between the joint moments calculated by the strain gauges (M’) and the joint moment estimated using the AMTI measurements (M) at each joint of the articulated leg using the set-up illustrated in Fig. 3. **b**) Bland and Altman plots showing the differences between joint moments as calculated by the strain gauges at the ankle (M_a_’), knee (M_k_’) and hip (M_h_’), and estimated with the AMTI force platform at the ankle (M_a_’), knee (M_k_’) and hip (M_h_’) against the average values (dotted line), with 95% limits of agreement (LOA; grey shadowing) for each of the eleven tests conducted for each joint

Figure 5-a illustrates the regression line between both methods of measurement. Determination coefficients between the two methods are equal to R^2^ = 0.9985–0.9998 for the three joints. The regression equations are as follows: y = 1.08x + 0.20 for the ankle, y = 1.03x + 0.22 for the knee, and y = 0.99x + 0,05 for the hip.

Agreement between the measurements is illustrated in Fig. 5-b using Bland and Altman plots. For the ankle joint, the bias was 0.746 (CI = 0.538–0.954) with a lower limit of agreement (LOA-) equal to 0.139 (CI = − 0.227–0.506) and LOA+ equal to 1.352 (CI = 0.985–1.719). The difference plot allowed the authors to evaluate a positive trend of differences, proportional to the magnitude of the measurement. The bias became greater as the joint moment increased. For the knee joint, the bias was 0.602 (CI = 0.451–0.753) with LOA- equal to 0.160 (CI = − 0.107–0.427) and LOA+ equal to 1.043 (CI = 0.776–1.310). The difference plot allowed the authors to evaluate a low positive trend of differences, slightly proportional to the magnitude of the measurement. The bias became greater as the joint moments increased. For the hip joint, the bias was − 0.128 (CI = − 0.227– − 0.029) with LOA- equal to − 0.417 (CI = − 0.591– − 0.242) and LOA+ equal to 0.161 (CI = − 0.014–0.335). The difference plot allowed the authors to evaluate a negative trend of differences, not proportional to the magnitude of the measurement.

There was a significant bias for all three joints since the line of equality was not present in the bias CI. The LOAs, if expressed as a percentage of the mean joint moment measurements, were as follows: 15.6% for the ankle, 10.0% for the knee and 8.5% for the hip. The variance of the difference was not influenced by the size of the measurement; hence, heteroscedasticity was absent in all tests.

### Assessment of dynamometry efforts

**Fig. 6:**
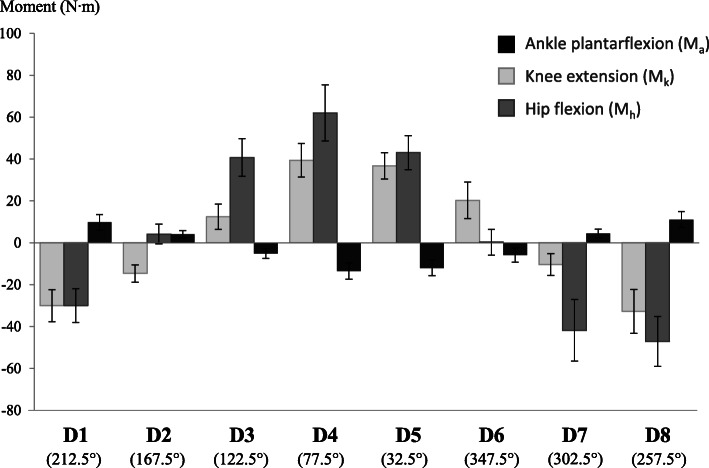
Joint moments for the hip, knee and ankle averaged among the 5 subjects during efforts in the eight directions (D1 to D8). Standard deviations are indicated by a bar. Positive values indicate plantarflexion of the ankle, knee extension and hip flexion

Figure 6 illustrates mean joint moments generated by subjects in the different directions tested. Each joint moment demonstrates a sinusoidal change across directions. Moment amplitudes for each joint differ in each direction. For the hip, mean joint moments varied between -47.12 N·m (extension) for D8, and 62.04 N·m (flexion) for D4. Hip joint moments were smaller for D2 and D6. For the knee, mean joint moments varied between − 32.78 N·m (flexion) for D8 and 39.38 N·m (extension) for D4. Knee moments were smaller for D2, D3 and D7. For the ankle, mean joint moments varied between − 13.52 N·m (dorsiflexion) for D4 and 11.05 N·m.(plantarflexion) for D8. Ankle moments were smaller for the D2 and D3 directions.

### Patterns of muscular activity

**Fig. 7:**
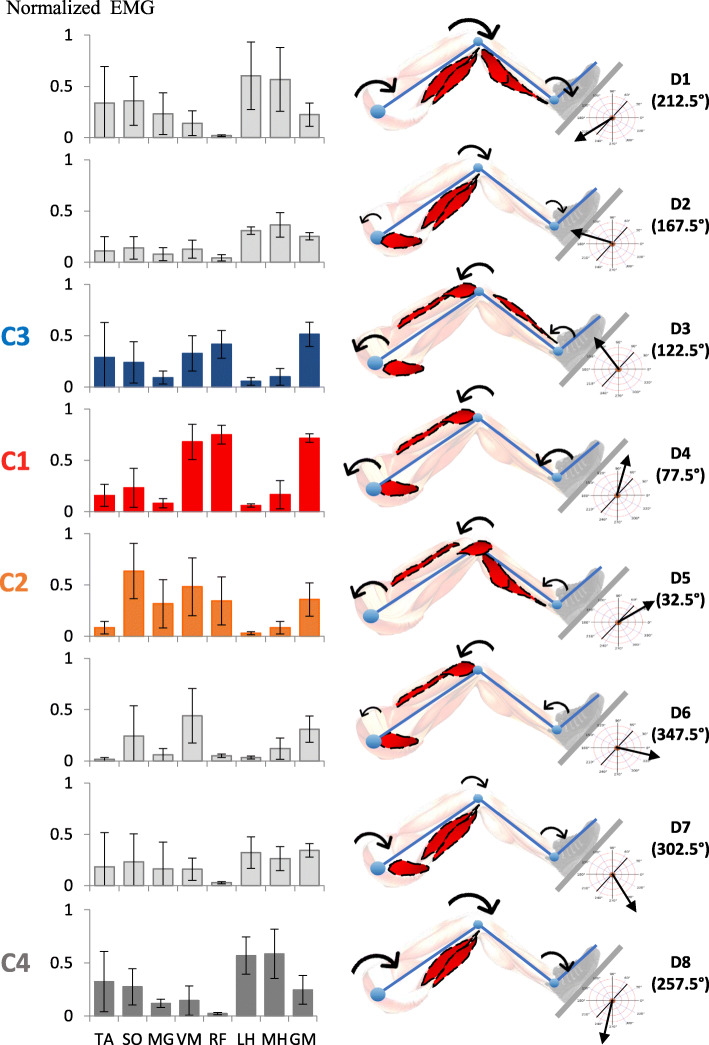
Normalized RMS values of the EMG during progressive efforts in the eight directions with the corresponding LL joint moment directions and predominant muscular activity. Standard deviations are indicated by a bar. Four muscle synergies were previously identified during gait: synergy 1 includes activity of the VM, RF and GM (red); synergy 2 includes activity of MG and SO (orange); synergy 3 includes activity of TA and RF (blue) and synergy 4 includes activity of LH and MH (grey)

The normalized RMS muscle activity values recorded for the eight LL muscles were calculated during the directional efforts and are presented in Fig. 7, also with the corresponding joint moment directions and predominant muscular activity observed during the dynamometry efforts assessment. Levels of activity of a given muscle were modified according to the direction of effort. Different patterns of muscular activity emerged for all directions of efforts, except for D1 and D8 which had relatively similar muscle activity patterns.

The cosine similarity between the muscular activity during pushes in the different directions and the four (C1, C2, C3 and C4) muscular synergies previously found during gait [12] are shown in Table 2. The muscular synergy C1 represented by the VM, RF and GM had the highest cosine similarity in the D4 direction. The muscular synergy C2 represented by the MG and SO had the highest cosine similarity in the D5 direction. The muscular synergy C3 represented by the TA and RF had the highest cosine similarity in the D3 direction. The muscular synergy C4 represented by the LH and MH had the highest cosine similarity in the D8 direction.

**Table 2:** Cosine similarity between muscles electromyography measured for the eight different push directions and the muscle synergies weight previously measured during gait.

Synergy	Push direction							
	D1	D2	D3	D4	D5	D6	D7	D8
C1	0.501	0.663	0.931	0.973^a^	0.784	0.874	0.721	0.522
C2	0.549	0.461	0.488	0.369	0.789^a^	0.515	0.619	0.451
C3	0.423	0.377	0.755^a^	0.641	0.459	0.313	0.446	0.425
C4	0.902	0.899	0.34	0.317	0.251	0.377	0.78	0.933^a^

^a^Highest cosine similarity for each muscle synergy during gait

## Discussion

### Joint moments measured by the apparatus are valid

Known joint moments applied by the instrumented leg were correctly calculated using the apparatus indicating that the static equations were appropriately implemented in the software. Pearson correlation coefficients showed a strong relationship between the moments applied by the instrumented leg and those estimated by the apparatus using the AMTI force platform measurements. The results of the Bland and Altman analyses demonstrate a positive bias for the ankle and knee joints, and a negative bias for the hip joint. The absence of the line of equality in CI for the three joints suggests a significant systematic difference between both moment estimations [27]. The significant bias was less than 0.75 N·m for the ankle, knee and hip, which was determined a priori as acceptable (less than 1 N·m). For example, mean joint moments observed during progressive efforts were 7.73, 23.14 and 32.0 N·m respectively for the ankle, knee and hip. The plots for the ankle and knee indicate proportional difference variability between measurements (i.e. constant coefficient of variation across the range of concentration). This is probably the result of a calibration error with one of the transducers or a greater lever arm measurements error made at the joints when computing the final results from the AMTI force platform measurements [27]. Moreover, the limits of agreement represented 8.5, 10.0 and 15.6% of the average joint moment calculated by both methods for the hip, knee and ankle respectively. In our opinion, these results suggest acceptable agreement between the two methods.

### Joint moments and EMG recordings change according to effort of direction

The results indicate that joint moments and patterns of muscular activity recorded during progressive static efforts on the apparatus change according to the direction of effort. In general, individual muscle shows sinusoidal activity across directions as expected during isometric efforts in different directions [28]. For example, the GM is fully activated in directions C3 and C4 and less activated in the other directions. Similarly, the VM is activated in directions D4 and D5, but proportionally decreases in activity as it deviates from these directions.

As demonstrated in Fig. 7, patterns of muscular activity are observed for a specific direction of effort. This confirms the hypothesis that effort at 50% of the maximal force in different directions allows for specific muscle patterns of activation. However, in some directions, the variability of muscle activation suggests that subjects can use different patterns of muscular activity. As an example of a different co-contraction strategy, the high standard deviation for both the calf and hamstring muscle while pulling the foot downward (D1, D7 and D8 directions) suggests that subjects can use either the calf or hamstring muscle to share the effort in the downward direction. For this example, the methodology could be improved by providing feedback from the ankle and knee joint moments to predominantly recruit calf or hamstring muscles.

### Joint moments and muscular activity pattern similarities during gait

The results suggest that there are some similarities between the joints moments measured for two of the eight dynamometry effort directions and the joint moments previously identified during gait for specific portions of the gait cycle [29, 30]. The results reported in Table 2 also suggest that there are similarities between the patterns of muscular activity for four specific directions of effort assessed with the dynamometer and the synergistic muscular activity patterns previously identified during gait among healthy individuals for some specific portions of the gait cycle [12, 31, 32].

First, during pushes in the D4 direction at 77.5 degrees, hip flexion, knee extension and ankle dorsiflexion moments with the muscular activity of the VM, GM and RF were observed. These measures partially characterize early stance phase (0–12%) moments, where hip extension moments should have been measured. These measures clearly characterize C1 muscular activity patterns related to weight absorption following heel strike. The hip extension moment with EMG activation of the GM and RF muscles could be improved by requiring subjects to push down and back with their foot and by providing feedback to elicit specific hip and knee extension moments.

Second, during pushes in the D5 direction at 32.5 degrees, hip flexion, knee extension and ankle dorsiflexor moments with the muscular activity of the SO and MG were observed. These measures partially characterize terminal stance phase (30–50%) moments, where plantarflexion moments should have been measured. These measures characterize relatively well C2 muscle synergy related to the forward propulsion by the triceps surae muscle. Plantarflexion moments with a higher proportion of MG EMG activity should have been measured to better replicate the terminal stance phase. Kinetic and EMG measures could be improved by providing feedback on joint moments exerted by the ankle and requiring specific plantar flexion.

Third, during pushes in the D3 direction at 122.5 degrees, hip flexion, knee extension and ankle dorsiflexion moments with the muscular activity of the RF and TA were observed. These measures characterize relatively well initial swing phase (62–75%) moments and C3 muscle synergy related to leg forward acceleration. EMG activation of the RF and TA muscles could be improved by requiring subjects to kick a ball with their foot on a virtual platform and by providing feedback to elicit specific knee extension and ankle dorsiflexion moments.

Fourth, during pushes in the D8 direction at 257.5 degrees, hip extension, knee flexion and ankle plantar moments, with the muscular activity of the LH and MH were observed. These measures clearly characterize terminal swing phase (87–100%) moments and C4 muscle synergy related to leg forward deceleration prior to heel strike.

### Potential of the methodology to be incorporated into a rehabilitation program

A rehabilitation program using this methodology could be used to train muscular activity patterns identified during gait using four (D3, D4, D5 and D8) of the eight directions identified. This methodology also has the potential to provide feedback about joint moments during progressive, static, directional efforts to replicate precise joint moments previously described during gait. This methodology could be improved by providing feedback on joint moments exerted at the ankle, knee and hip to better replicate gait kinetics and EMG during the early stance, terminal stance and initial swing phases of gait. Such a program could improve both poor coordination and weakness of specific muscle groups [33]. Although some evidence suggests that such training could be conducted and improve gait [16], no studies have investigated whether people post-stroke would be able to exert and control these directional efforts to use the apparatus or whether such a training program could translate into improvement of functional activities such as gait.

### Limitations

A potential limitation of the instrumented set-up was the use of a small force plate. Calculation of a joint moment requires the location of the center of pressure to be estimated in real time. The location of the center of pressure is based on the joint moment in the X axis and the force values in Y and Z axes. Since the length of the foot exceeds the length of the force plate, the force in the Z-axis could be applied outside the surface of the AMTI force plate. Although this does not seem to affect the measurements due to low forces being applied, a larger force plate would still be recommended.

An additional limitation of the instrumented set-up were angle and lever arm measurement errors. Although subjects had both their trunk and foot firmly fastened to the apparatus, the different muscle group contractions during efforts altered the joint angles leading to measurement errors. Using motion-capture data to improve estimation of each joint’s center of rotation and better monitor joint angles would be recommended.

Another limitation involves the study’s methodology given that the position on the static dynamometer does not reproduce the upright position during gait, neither the proprioceptive feedback related to the inertia of the LL segments or vestibular feedback related to body displacement associated with dynamic LL kinematics during locomotion. Hence, it is very important to understand that this methodology cannot directly be used for locomotion training. However, it could be used to train muscle coordination documented during gait in conjunction with gait training to optimize intensive rehabilitation functional achievements.

Finally, considering the small sample size and gender difference, it is not possible to generalize the results of the muscular activity patterns during directional pushes to a healthy or neurological population. A future study with a larger sample size study with healthy and post-stroke individuals is warranted to generalize and establish the inter-subject variability of the results.

## Conclusion

This research describes a new methodology that was shown to provide valid joint moments exerted at the LL. The results indicate that joint moments and patterns of muscular activity recorded during progressive static efforts on the instrumented apparatus are modified according to the direction of effort. For four of the eight directions, patterns of muscular activity were related to the data previously identified during gait. This methodology could be improved by providing feedback on joint moments exerted at the ankle and knee to better replicate gait kinetics and EMG during the initial stance, terminal stance and initial swing phases of gait. It is suggested that this methodology has the potential to recruit and train patterns of muscular activity impaired in stroke patients in addition to conventional training to optimize intensive rehabilitation functional achievements.

## Data Availability

Please contact the corresponding author for data requests.

## Abbreviations

BF: Biceps femoris
CI: Confidence interval
CNS: Central nervous system
CRIR: Centre for interdisciplinary rehabilitation research of greater montreal
EMG: Electromyography
GM: Gluteus medius
LL: Lower limb
LOA: Limit of agreement
MG: Medial gastrocnemius
NNMF: Non-negative matrix factorization
r: Pearson correlation
R^2^: Determination coefficient
RF: Rectus femoris
RMS: Root mean square
RMSE: Root mean square error
SD: Standard deviation
SO: Soleus
ST: Semitendinosus
TA: Tibialis anterior
VM: Vastus medialis
